# Reviews

**Published:** 1891-04

**Authors:** 


### EDITORIAL DEPARTMENT.

### REVIEWS.

The object of these columns will be to review briefly the principle works published in various other languages on veterinary medicine and the sciences pertaining to it.

For the full development of a science nothing is so absolutely necessary as the dissemination of the "latest" ideas and discoveries. Daily we hear of "new treatments" which on examination are found to have existed years before. The experimentalist likewise often duplicates work and only because of an inadequate knowledge of the literature pertaining to the subject. Do not infer, however, that the above is intended to be an intimation that in the realm of veterinary literature there is an overproduction. In no science are "knacks" treated with so much secrecy as in veterinary medicine, and especially is this true of veterinary medicine in America.

It is the duty of every physician to observe and record the progress of every interesting case, to note the effects of a particular treatment and to give the results to the profession. In this way only can harmony be achieved in veterinary medicine.

One idea can be gotten of the important role that the recording and preservation of observations pays, when we recognize that with the destruction of all recorded facts the sciences would be plunged into a state paralell to that in which they were thousands of years ago.

The sciences and in fact all the pursuits and functions which together make higher civilization began with the recording and dissemination of observations; they would degenerate and finally cease if this were no longer practiced.

The reviews from German journals in the present number are from the pen of Leo. Breisacher, V. M. D., whose presence in Germany, at the great Veterinary centre of Berlin, assures us of his cognisance of the most recent work there.

